# Long-term outcomes of surgical treatment for paediatric acute mastoiditis: the role of mastoidectomy

**DOI:** 10.1007/s00405-024-09072-3

**Published:** 2024-12-01

**Authors:** Matija Švagan, Janez Rebol

**Affiliations:** Department of Otorhinolaryngology–Head and Neck Surgery, University Clinical Center Maribor, Ljubljanska 5, 2000 Maribor, Slovenia

**Keywords:** Acute mastoiditis, Mastoidectomy, Long-term consequences, Paediatric otorhinolaryngology

## Abstract

**Purpose:**

Despite the declining incidence of acute mastoiditis (AM) due to antibiotics, complications persist, necessitating surgical intervention in severe cases. Recent studies suggest conservative treatments, avoiding mastoidectomy, show high recovery rates. However, this trend raises concerns about severe complications, prolonged treatment, increased antibiotic use, and declining surgical skills. While much research focuses on AM pathogenesis and treatment, the long-term consequences, especially post-mastoidectomy ear function, are less understood. To address this, we studied the permanent effects of surgically treated AM and mastoidectomy on ear function.

**Methods:**

A cohort of patients that received surgical treatment for AM in the form of mastoidectomy was invited to be tested after at least 5 years since the operation. Test battery included COMQ-12 questionnaire, physical exam and otomicroscopy, extended high pure tone audiogram, DPAOE and middle ear impedance testing. Results were compared with a control group and a group which received surgical treatment in the form of tympanostomy for acute otitis media with impeding mastoiditis.

**Results:**

The COMQ-12 questionnaire yielded higher scores in questions about hearing in quiet environments, hearing in noise, tinnitus, and ear discomfort. Minor structural changes were observed in the test groups during otomicroscopy, but not in the control group. Pure tone audiometry revealed a median elevation of around 10 dB in high and extended high frequencies, with similar results observed in DPAOE testing. In middle ear impedance testing, only an elevation of the stapedial reflex threshold was noted; other tests did not show any statistically significant differences.

**Conclusions:**

In the long term, the majority of patients post-AM have minor functional in structural consequences. In the context of treatment of AM, the effects of mastoidectomy are negligible when compared to less invasive surgical procedures.

**Supplementary Information:**

The online version contains supplementary material available at 10.1007/s00405-024-09072-3.

## Introduction

Despite the widespread use of antibiotics, acute mastoiditis (AM) and its associated complications persist, although their incidence is declining. Surgical intervention remains crucial, especially in cases involving intracranial complications and microbial resistance [1–7]. Recent years have seen an increase in studies exploring conservative treatment methods for AM, primarily avoiding mastoidectomy or avoiding surgical therapy altogether. These studies report remarkable recovery success; however, the trend to reduce the frequency and scope of surgical drainage operations raises concerns about the higher risk of severe complications of AM, prolonged treatment duration, increased use of antibiotics, and a decrease in surgical proficiency and knowledge [4, 6, 8–14]. While a substantial body of literature is devoted to the pathogenesis and treatment of acute mastoiditis, less is known about the long-term consequences of AM, particularly the effects of surgical removal of the mastoid cell system [15–17]. To address these gaps, we conducted a study to investigate the permanent consequences of surgically treated AM and the effect that mastoidectomy has on ear function.

## Methods

The study was approved by the National Medical Ethics Committee of the Republic of Slovenia (0120-547/2021/3). It was conducted in accordance with the Declaration of Helsinki, and all participants provided informed consent prior to their inclusion in the study.

### Patient selection

A retrospective review of medical records was conducted for children who underwent surgical treatment for acute mastoiditis between July 2001 and October 2019 at the Department of Otorhinolaryngology, University Clinical Center of Maribor. Over the 18-year period, the surgical indications for mastoidectomy remained consistent and included otalgia, fever, and postauricular swelling. All procedures were performed by the same surgeons under general anesthesia. The surgical technique involved making an incision over the mastoid region, drilling the cortical bone, and opening the major air cells. Access to the epitympanum was ensured, and drainage through the tympanic membrane was facilitated by placing a tympanic tube. A drainage tube was also placed in the mastoid cavity, and the incision was closed in two layers. Some patients in the cohort had simultaneous contralateral purulent acute otitis media (AOM), characterized by otalgia and tympanic membrane lateralization, indicating incipient mastoiditis. Tympanostomy tubes were inserted for drainage on the affected contralateral side as well.

Out of 109 patients who underwent mastoidectomy for AM, contact information was available for 97. Of these, 67 met the additional criterion of undergoing mastoidectomy with tympanostomy for AM on one side, as well as contralateral tympanostomy for concurrent AOM with incipient AM. These patients were invited to participate in the study, and 30 responded. Additionally, 30 volunteers with no history of ear disease or hearing complaints were randomly selected to form a control group. Both groups followed the same study protocol. For the analysis, it was decided to split the data by ear, resulting in three groups: the tympanostomy and mastoidectomy group (Group TM—30 ears), the tympanostomy group (Group T—30 ears), and the control group (Group C—30 ears).

### Review of medical records and examination

A review of the medical records of operated patients was conducted first. Otomicroscopy was performed using a standard office microscope. To facilitate analysis and ensure objectivity, a grading system was designed. Pathology was categorized and graded using a Likert scale. Atrophy, thickness and scarring of the tympanic membrane, myringosclerosis and myringitis were graded from 0 to 3. Grade 0 indicated the complete absence of pathology, grade 1 indicated pathology present on less than one-quarter of the eardrum, grade 2 indicated pathology present on less than half of the eardrum, and grade 3 indicated pathology present on more than half of the eardrum. Retraction of the tympanic membrane was also assessed and graded from 0 to 4 according to the classification of epitympanic retractions by Toš and pars tensa retractions by Sadé [18, 19].

**Fig. 1:**
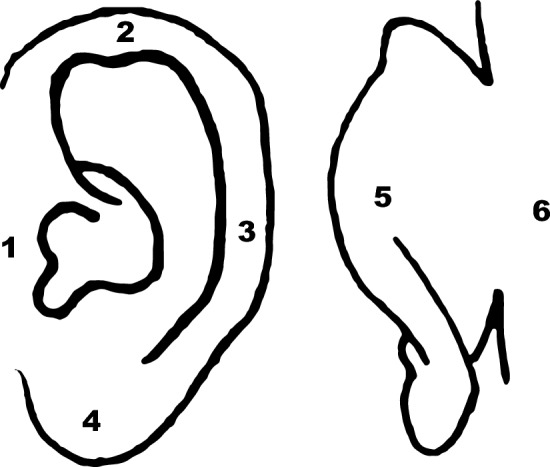
Sensory testing locations

Sensory testing of the auricle was performed using a size 1.65 Semmes–Weinstein monofilament on six different locations on the auricle, as illustrated in Fig. 1 [20].

### COMQ-12 questionnaire

The Chronic Otitis Media Questionnaire 12 (COMQ-12) is a 12-item multiple-choice disease-specific health-related quality-of-life (HRQoL) questionnaire [21]. Each question has a Likert scale from 0 to 5, depending on severity. Translation, cross-cultural adaptation, and validation were completed prior to this study [22]. Whole questionnaire is attached in the Supplementary Information. Questionnaire was filed out by both, patients with mastoidectomy and tympanostomy, as well as by the participants in the control group.

### Audiometry

Extended high pure tone audiograms (125 Hz–20 kHz) were conducted using the Interacoustics AC40 Clinical audiometer by the same experienced audiometrist. For easier interpretation results of audiometry were divided into 4 average values: low tone average (< 500 Hz—LTA), middle tone average (0.5–4 kHz—MTA), high tone average (4–8 kHz—HTA) and extended high tone average (8–20 kHz—EHTA). For calculating middle tone average same method as widely accepted method of pure tone average (PTA) was used [23].

### Middle ear impedance testing

Single band (256 Hz) tympanometry was performed with the Interacoustics AT235 tympanometer. Values of external auditory canal volume, gradient, tympanometric peak pressure, and compliance were recorded. The lowest elicitable ipsilateral stapedial reflexes at frequencies 500, 1000, 2000, and 4000 Hz were also recorded with the same equipment. All measurements were repeated twice; the average value was recorded for continuous values, and for stapedial reflexes, the lowest value was recorded.

Wide band tympanometry was performed with the Interacoustics Titan hardware. Wide band values of external auditory canal volume, tympanometric peak pressure, compliance, and middle ear resonant frequency were recorded. All measurements were repeated twice, and average values were recorded.

### Distortion-product otoacoustic emissions (DPAOE)

Distortion-product (DP) otoacoustic emissions were recorded using Interacoustics Eclipse hardware. The stimulus level was set to 65 dB for frequency 1 (f1) and 55 dB for frequency 2 (f2), with an f2/f1 ratio of 1.22, test duration of 60 s, and minimum reliability of 98%. Values of DP level, noise, signal-to-noise ratio, and reliability were recorded. DP levels were measured twice on 12 f2 frequencies ranging from 500 Hz to 10 kHz. Measured values were intensity corrected for the level of noise and an average of two series was recorded. Only DPAOE that passed reliability of 98% were taken into account, in case the emissions detected had a lower reliability. With a similar method to pure tone audiometry frequency averages were calculated for low, medium and high frequencies.

### Data analysis

The statistical analysis was performed using the statistical package SPSS V29. Descriptive statistical methods were used for sample description. Results are presented as median values with 95% confidence intervals or frequencies and percentages. The Shapiro–Wilk test was used to confirm non-normal data distribution. Comparisons between groups were analyzed using two-sided Chi-square (χ^2^) tests, Kruskal–Wallis Tests, Independent Samples Median Tests, and Spearman Correlation. Results were considered statistically significant for *p*-values below 0.05.

## Results

The median age at the time of presentation was 2.1 years, with the youngest patient undergoing surgery at 0.64 years and the oldest at 11.3 years. A history of AOM was recorded in 10% (*n* = 3) of the patients, with 3.3% (*n* = 1) having experienced two episodes of AOM prior to AM. The remaining patients had no documented history of acute ear infections. No preexisting hearing loss was reported by the parents before the onset of AM. Additionally, 96.7% (*n* = 29) of the patients underwent neonatal hearing screening, and all passed. Prior to presentation, 30% (*n* = 9) of patients received systemic oral antibiotic treatment, no local treatment was recorded. The signs reported by the attending physician for the TM group were as follows: severely lateralized eardrum in 96.7% (*n* = 29) of patients, bony part ear canal edema in 66.6% (*n* = 20), retroauricular redness and swelling in 100% (*n* = 30), retroauricular fluctuation in 23.3% (*n* = 7), and otorrhea in 26.6% (*n* = 8). For the T group, the reported signs included: severely lateralized eardrum in 100% (*n* = 30), bony part ear canal edema in 23.3% (*n* = 7), retroauricular redness and swelling in 0% (*n* = 0), retroauricular fluctuation in 0% (*n* = 0), and otorrhea in 30% (*n* = 9). The median duration of symptoms prior to surgery was 3 days, ranging from 0 to 11 days. The median white blood cell count was 14.25 × 10^9^/L (range: 2 × 10^9^/L to 35 × 10^9^/L), and the median C-reactive protein level was 99 mg/L (range: 5–280 mg/L). A preoperative CT scan was performed in 16.7% (*n* = 5) of cases. *Streptococcus pneumoniae* was the predominant pathogen, isolated in 57% (*n* = 17) of cases, followed by *Streptococcus pyogenes* (Group A) in 20% (*n* = 6). *Turicella otitidis*, *Pseudomonas aeruginosa*, and *Staphylococcus aureus* were each isolated in one case. In 10% of cases, the swabs were sterile. Swabs were collected bilaterally, and the same pathogen was isolated on both sides, except for *Pseudomonas aeruginosa*, which was detected only on the side where mastoidectomy was performed.

The median age of all groups was 14.3, 14.1, 14.1 and 14.5 years for groups TM, T and C, respectively. The median hospital stay was 10.5 days, ranging from 5 to 78 days. The median duration of retroauricular drainage placement was 9.5 days, with a range of 4–20 days.

In the tested group, 3.3% (*n* = 1) patients presented with concurrent meningitis, 3.3% (*n* = 1) with sigmoid sinus thrombosis and 3.3% (*n* = 1) with labyrinthitis, but no surgical complications were reported. Among the 109 mastoidectomies performed during the observational period, complications were recorded as follows: 4.5% (*n* = 5) cases of labyrinthitis, 2.8% (*n* = 3) cases of meningitis, 2.8% (*n* = 3) cases of facial nerve paresis, 1.8% (*n* = 2) cases of sigmoid sinus thrombosis, 1.8% (*n* = 2) cases of sepsis, and 0.9% (*n* = 1) case of a cranial base defect involving the posterior fossa. Only two surgical complications were observed: excessive bleeding due to an undiagnosed coagulation disorder and retroauricular skin necrosis. After surgery patients were monitored for a median period of 13 months (range: 7–37 months). During this follow-up period, 20% (*n* = 6) of the patients experienced recurrent bilateral AOM following the initial surgery, and 10% (*n* = 3) required repeat bilateral tympanostomy. One patient (3%) developed an eardrum perforation on the side of the AM, which was successfully repaired with myringoplasty. After the follow-up period, all patients remained asymptomatic, with no reported events—either by the patients or their parents—that could have affected hearing function. The median time from surgery to the execution of this study’s test protocol was 11.61 years (range: 4.98–22.07 years).

All participants in Group C answered every question on the COMQ-12 questionnaire with a score of 0. Since the questionnaire is not ear-specific, Group TM and Group T were combined into a single test group for this analysis. The results of the COMQ-12 questionnaire for the test group are summarized in Table 1. Significant differences between the test and control groups were observed for questions 3, 4, 5, and 7 which address hearing in quiet environments, hearing in noise, discomfort around the ear, and tinnitus, respectively. For a detailed description of the questionnaire items, please refer to the Supplementary Information.

**Table 1:** Score averages, distributions and comparisons for individual COMQ-12 question responses

Question no	1	2	3	4	5	6	7	8	9	10	11	12
Mean	0.20	0.07	**0.50**	**0.93**	**0.33**	0.27	**0.47**	0.00	0.07	0.20	0.07	0.20
Median	0.00	0.00	**0.00**	**0.00**	**0.00**	0.00	**0.00**	0.00	0.00	0.00	0.00	0.00
St. deviation	0.61	0.365	**1.009**	**1.258**	**0.711**	0.691	**0.900**	0.000	0.365	0.761	0.365	0.484
Variance	0.37	0.133	**1.017**	**1.582**	**0.506**	0.478	**0.809**	0.000	0.133	0.579	0.133	0.234
Minimum	0	0	**0**	**0**	**0**	0	**0**	0	0	0	0	0
Maximum	3	2	**4**	**4**	**3**	3	**3**	0	2	3	2	2
Statistic (χ2)	2.62	0.001	**7.57**	**13.41**	**6.25**	2.87	**7.57**	0.000	7.57	0.586	0.001	2.67
Significance	0.11	0.97	**0.006**	** < 0.001**	**0.012**	0.62	**0.006**	> 1.0	0.974	0.444	0.974	0.067

Statistically significant differences are shown in bold

Sensory testing revealed that 10% (3) of patients had reduced sensitivity on location 5, 6.6% (2) on location 6 and 3.3% (1) on location 4, otherwise no sensory deficit was detected, as it was not detected in control groups and contralateral ears.

Otomicroscopic exam scoring results are summarized in Table 2. No significant differences were found between groups T and TM. Both group T and TM had significantly higher score than group C in all observed areas except retraction of pars tensa and myringitis.

**Table 2:** Median values of the grades allocated during the otomicroscopy examination

	Atrophy	Thickening and scarring	Myringosclerosis	Myringitis	Retraction (Toš)	Retraction (Sadé)
TM	0.4	0.27	0.63	0.03	0.43	0.10
T	0.57	0.27	0.63	0.03	0.03	0.03
Control	0	0	0	0	0	0
*F* (*p*)	19.066 (< 0.001)	5.984 (0.05)	17.318 (< 0.001)	1.090 (0.580)	12.539 (0.002)	2.182 (0.336)

**Fig. 2:**
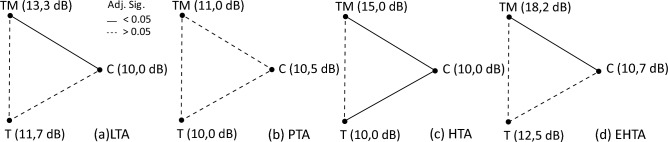
Median values of audiometric frequency averages compared between the groups: **a** low tone average, **b** middle/pure tone average, **c** high tone average, **d** extended high tone average

Median values of audiometric frequency averages are presented in Table 3. There were no significant differences in the PTA values among the three groups. However, significant differences were observed when comparing Group TM with Group C in LTA (*p* =0.033), HTA (*p* < 0.001) and EHTA (*p* =0.007). When comparing Group T to Group C, significant differences were found only in HTA (*p* =0.015), while no significant differences were observed in all other frequency averages. No significant differences were found when comparing Group TM and Group T. The results are presented graphically in Fig. 2.

**Table 3:** Median values of audiometric frequency averages

		Control	Timpanostomy	Timpanostomy and mastoidectomy
LTA (dB)	Median	**10.0**	**11.7**	**13.3**
	Std. dev	2.0	3.8	5.9
	Min	10.0	10.0	10.0
	Max	16.7	25.0	31.7
PTA (dB)	Median	**10.5**	**10.0**	11.0
	Std. dev	2.1	3.2	6.5
	Min	10.0	10.0	10.0
	Max	19.0	21.8	35.0
HTA (dB)	Median	**10.0**	**10.0**	**15.0**
	Std. dev	0.7	5.7	19.3
	Min	10.0	10.0	10.0
	Max	13.3	30.0	108.3
EHTA (dB)	Median	**10.7**	**12.5**	**18.2**
	Std. dev	4.3	9.6	21.3
	Min	7.11	9.3	8.6
	Max	25.0	43.6	120

Most important values are shown in bold

When comparing the results of the COMQ-12 questionnaire and pure tone audiometry, we found that the EHTA results were significantly correlated with responses about worsened hearing in quiet (ρ = 0.480, *p* =0.037), worsened hearing in noise (ρ = 0.424, *p* =0.019), and the perception of tinnitus (ρ = 0.392, *p* =0.032). Significant correlations were also found between EHTA and the number of symptomatic days until surgical treatment (ρ = 0.413, *p* =0.023), and a near-significant correlation was observed between HTA and symptomatic days until surgical treatment (ρ = 0.334, *p* =0.071). Additionally, a significant correlation was identified between the number of symptomatic days and the perception of worsened hearing in noise (ρ = 0.384, *p* =0.036) as measured by the COMQ-12 questionnaire.

**Fig. 3:**
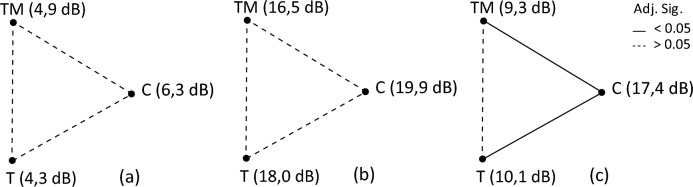
Comparison of median noise-adjusted DPAOE levels between groups: **a** low tone average, **b** middle/pure tone average, **c** high tone average

Noise-adjusted DPAOE levels were correlated with pure tone audiometry averages at the same frequencies (LTA, MTA, HTA), and all correlations were significant (*p* < 0.005). DPAOE levels showed no significant differences between the groups at frequencies at LTA, MTA and between groups T and TM in HTA. Significant difference was found in HTA between groups TM and C (*p* =0.011) and T and C (*p* =0.033). For a graphical representation, please refer to Fig. 3.

**Fig. 4:**
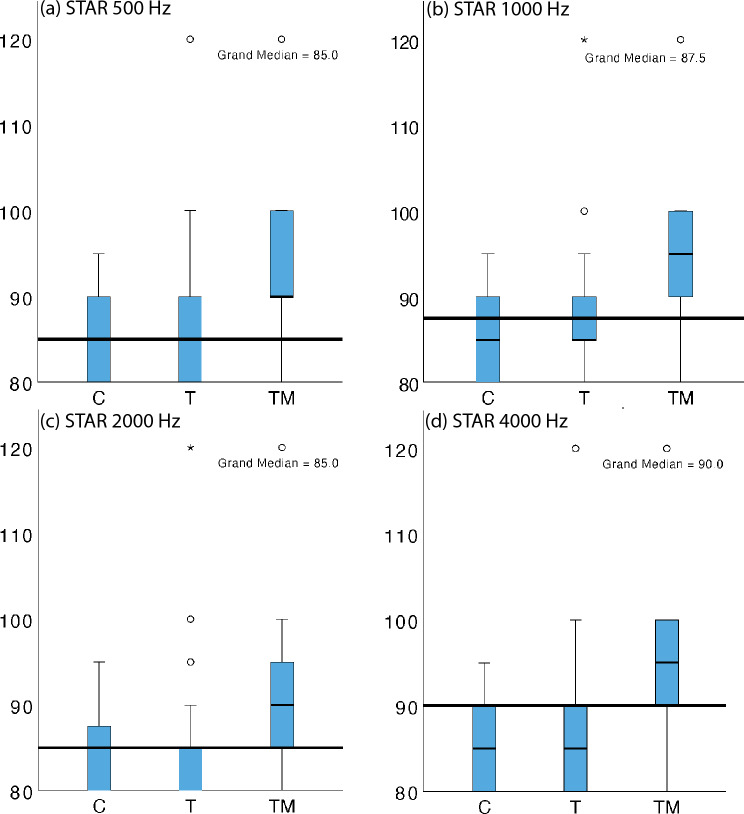
Comparison of acoustic reflex threshold values between groups

Tympanometry at 256 Hz and wideband tympanometry demonstrated that Group C had a significantly larger external ear canal volume (1.1 ± 0.24 ml) compared to Group TM (0.86 ± 0.19 ml) and Group T (0.9 ± 0.2 ml). Median tympanometric peak pressures, admittance at 256 and 1000 Hz, and middle ear resonant frequencies were all within normative values. Differences between Groups TM, T, and C in these measurements were statistically insignificant (*p* > 0.05). However, ipsilateral stapedial reflex testing at four frequencies revealed that reflex thresholds were significantly elevated in Group TM compared to Groups T and C (*p* < 0.001) (Fig. 4). For detailed data, please refer to the table in the Supplementary Information.

## Discussion

This study aimed to determine the long-term effects of mastoidectomy for treatment of AM in paediatric patients. Although incidence of AM has declined, it remains a serious complication of AOM, and rising antibiotic resistance raises the question of medical versus surgical management [7, 24]. As evidence grows for conservative treatments, it’s crucial to compare outcomes of mastoidectomy with other options to support surgical intervention [9–11]. Diagnostic ambiguity and lack of international consensus on mastoidectomy criteria in AM lead to variability in treatment decisions. Additionally, confirmation bias may influence the decision to perform surgery in very ill children with AM, as surgeons may prefer surgical intervention to avoid the risk of sequelae or mortality in young patients. Conversely, the negative implications of performing an unnecessary mastoidectomy, when less invasive treatment might suffice, must also be considered. This variability impacts both treatment decisions and recovery rates [25, 26]. Studying AM bares inherent challenges due to infeasibility of blinded prospective studies in life-threatening conditions. When evaluating the sequelae of AM treated with mastoidectomy one must consider two factors: fulminant inflammation and the removal of cells that form part of an intricate aerated system. The loss of function due to cell removal is not yet fully understood.

The cohort examined in this study is unique as it allows for a comparative analysis of the effects of a more invasive surgical intervention on one side and a less invasive procedure on the contralateral side within the same patient. Patients who underwent mastoidectomy were diagnosed AM based on widely accepted diagnostic criteria, including otalgia, fever, and postauricular swelling. Conversely, on the side where only tympanostomy was performed, mentioned diagnostic criteria of AM were not met; however, symptoms of purulent AOM such as lateralization of the tympanic membrane, oedema of the posterior wall of the external auditory canal were observed indicating possible progress to AM. Pathophysiologically, mastoiditis is likely present in all instances of AOM, particularly in more severe cases necessitating tympanostomy. A limitation of this study is the inability to retrospectively assess the differential extent of inflammation on each side, which may lead to an overestimation of the sequelae attributed to mastoidectomy.

Comparing our study cohort to similar studies in demographics, hospital stay, laboratory and microbiological findings, as well as complication rates showed consistent results, except for a longer hospital stay in our cohort [15, 17, 27, 28]. The discrepancy in recorded hospital stays arises from our institution’s practice of officially recording paediatric patients as hospitalized during the acute phase, even though they may be discharged and return for follow-up checkups, thus formally extending the recorded duration of hospitalization. As a result, the actual inpatient duration cannot be accurately calculated retrospectively. All participants who responded to the invitation were included in the study without further exclusions. However, some degree of selection bias may have occurred, as we could not control for participants’ motivations for responding. Additionally, the complication rate observed in the tested group was similar to that of all AM cases treated with mastoidectomy during the observational period. Despite these limitations, the similarities between our study group and larger studies suggest that our findings can be reasonably extrapolated to a larger population.

For the majority of patients, AM represented the first presenting acute infection of the middle ear. Only a few had documented episodes of AOM, no additional episodes were reported by parents. Due to the young age of the patients, objective assessment of hearing loss was limited to neonatal hearing screening tests. Since neonatal hearing screening has been routinely implemented in Slovenia since 2005, only one patient, who was older, did not undergo screening, all other patients passed the test. Based on this, we believe that preexisting hearing loss prior to the episode of AM is unlikely in any of the analysed patients. The only factor that could have potentially influenced long-term hearing outcomes was recurrent AOM in a subgroup of patients. However, these patients received appropriate treatment, making significant long-term hearing impairment unlikely.

Median pure tone audiometry thresholds of the control group at all frequencies were 10 dB. This value was thus considered the normal hearing threshold in the test groups. Taking the benchmark into accout it is evident that hearing loss overall is not substantial, with losses less than 10 dB.

Examining the data by group (Table 3, Fig. 2), it is apparent that variability increases significantly at higher frequencies, indicating a high-frequency hearing loss in a subset of patients. Comparing groups T and TM, no significant differences in hearing thresholds were observed, even in EHTA. The hearing thresholds of groups C and TM differed significantly in all averages except for the PTA, suggesting that although hearing loss is present, patients probably do not experience significant difficulties in speech comprehension. It is not possible to determine the extent of inner ear damage attributable to inflammation, but based on these findings, one might infer that mastoidectomy in the treatment of acute AM did not adversely affect hearing thresholds. On the contrary, our data suggest that it may reduce hearing loss if performed promptly.

The average scores for all questions in the COMQ-12 were below 1, indicating that, overall, patients who underwent surgery for acute mastoiditis do not exhibit symptoms indicative of chronic inflammation of the middle ear. However, significant differences were noted when comparing the responses of the test group to those of the control group, specifically in questions that address hearing in quiet environments, hearing in noise, discomfort around the ear, and tinnitus. This finding suggests that despite the low median and mean scores, a subset of patients experience difficulties in these areas. This is further supported by the significant correlation between high-frequency hearing loss and higher scores in questions that address hearing in quiet environments, hearing in noise and tinnitus. The higher score in question about discomfort around the ear can be attributed to sensory loss in the auricle. Temporary loss of sensation in the auricle following ear surgery is well-documented in the literature, but our findings indicate that up to 10% of patients experience some, albeit limited, permanent sensation loss in the auricle [20, 29, 30].

Although significant structural differences in eardrums were observed with otomicroscopy in both test groups compared to the control group, no significant differences were found between the test groups themselves. We acknowledge the subjective component of physical examinations, therefore, we utilized DPOAE and impedance testing to further assess middle ear function. DPOAE was employed because levels never return to normal after middle ear disease [31], and impedance testing was chosen due to its practicality and extensive knowledge base. Noise-adjusted DPOAE levels did not differ significantly between groups T and TM, but significant differences were observed between groups C and TM, and C and T at higher frequencies. These findings exhibit a pattern like that seen in pure tone audiometry. Despite the visual structural changes of the eardrum observed in otomicroscopy, tympanometry did not detect any significant differences between the groups in all measured values. In all groups, we observed lower median resonant frequencies of the middle ear, that one defined by the population average between 0.8 and 1.2 kHz. The resonant frequency in group C was 754 Hz, almost within the population average, but it was lower in groups T and TM at 722 and 667 Hz, respectively, indicating some damping due to mass effect in the affected middle ears [32]. However, none of these changes were statistically significant. Stapedial reflex testing detected a statistically significant elevation of thresholds in group TM at all tested frequencies. No such elevation was detected in groups C and T, with values remaining within previously published normative ranges [33]. The cause of the significant difference in the volume of external auditory canals between the test and control groups remains to be researched. Two possible explanations could be speculated: changes in the meatus due to scarring after surgery or changes in mastoid bone pneumatization after infection [34, 35].

Mastoidectomy does not appear to have contributed to structural changes in the tympanic membrane, nor does it alter middle ear biomechanics as measured by tympanometry. The elevated thresholds in acoustic stapedial reflexes found in group TM are notable as they represent the sole distinctive finding across the battery of tests. Given that no significant differences were detected in tests of inner ear function (pure tone audiogram, DPOAE), it can be assumed that this change is of a biomechanical nature affecting the ossicular chain.

## Conclusions

This study has demonstrated that, in the long term, the majority of patients post-acute mastoiditis experience hearing loss up to 10 dB in high frequencies, minor structural changes of the eardrum, and elevated stapedial reflex thresholds. These findings were also observed in the group that had fulminant AOM treated with tympanostomy alone. Therefore, in the context of AM treatment, the effects of mastoidectomy are negligible when compared to less invasive surgical procedures. Furthermore, it has been shown that prompt surgical treatment, including mastoidectomy, is more likely to prevent high-frequency hearing loss in AM.

## Supplementary Information

Below is the link to the electronic supplementary material.

Supplementary file1 (PDF 125 KB)

## Data Availability

Reference to the literature is included in the supplement. For other data availability statement is not applicable.
