# Supplementary material for: Long-term outcomes of surgical treatment for paediatric acute mastoiditis: the role of mastoidectomy
Source: Eur Arch Otorhinolaryngol. 2024 Dec 1;282(4):1815–23. doi: 10.1007/s00405-024-09072-3 (PMC11950107; doi:10.1007/s00405-024-09072-3)
Supplement: Supplementary file 1 — Supplementary file1 (PDF 125 KB) [file 405_2024_9072_MOESM1_ESM.pdf]

Supplemental Information: COMQ-12

Presented questions are presented here only for refference and easier understanding. For more information about COMQ-12 please refer to the original article:

Phillips, J. S., Haggard, M., & Yung, M. (2014). A new health-related quality of life measure for active chronic otitis media (COMQ-12): Development and initial validation. *Otology and Neurotology*, *35*(3), 454–458. https://doi.org/10.1097/MAO.0000000000000205

Symptom severity:

1.	Discharge or drainage from the ear	0	1	2	3	4	5
2.	Having a‘‘smelly ear’’	0	1	2	3	4	5
3.	Hearing problems at home, e.g. requiring the volume of the TV or radio to be turned up.	0	1	2	3	4	5
4.	Hearing problems when talking to people in groups or when there are noisy surroundings	0	1	2	3	4	5
5.	Discomfort in and/or around the ear	0	1	2	3	4	5
6.	Dizziness or feeling ‘‘off balance’’	0	1	2	3	4	5
7.	Tinnitus or noises in the ear	0	1	2	3	4	5

Lifestyle and work impact:

How often have you NOT been able to:

8.	Perform your normal daily activities at home / work?	0	1	2	3	4	5
9.	Wash or shower or bathe as you would like to? i.e., how often have you been fearful of these activities causing an ear infection?	0	1	2	3	4	5

Health service impact:

10.	How often have you been to see your GP about your ear problems?	0	1	2	3	4	5
11.	How often do you need to take medicines (including eardrops) for your ear problem?	0	1	2	3	4	5

General:

12.	To what degree do your ear problems ‘get you down’?	0	1	2	3	4	5

Supplemental Information: Middle ear impedance measurements

	Skupina K	Skupina T	Skupina TM	χ^2	p
Ear canal volume (ml)	1,1 (Q_1:0,91; Q_3:1,34)	0,91 (Q_1:0,78; Q_3:0,99)	0,86 (Q_1:0,67; Q_3:0,96)	14,6	**<0,001**
Tympanometric peak pressure (daPa)	-2,5 (Q_1:-10,75; Q_3:3,75)	-4 (Q_1:-19,0; Q_3:1,0)	-7 (Q_1:-28,0; Q_3:1,0)	0.84	0,65
Gradient	56,5 (Q_1:48,0; Q_3:73,0)	54,0 (Q_1:40,0; Q_3:53,5)	66,0 (Q_1:39,0; Q_3:79,5)	0,70	0,70
Admittance (256 Hz) (mmho)	0,56 (Q_1:0,36; Q_3:0,88)	0,63 (Q_1:0,47; Q_3:1,03)	0,63 (Q_1:0,33; Q_3:0,95)	0,44	0,81
Admittance (1000 Hz) (mmho)	2,5 (Q_1:1,0; Q_3:3,15)	1,2 (Q_1:0,6 Q_3:2,6)	1,2 (Q_1:1,2; Q_3:1,9)	6,0	0,051
Middle ear resosnt frequency (Hz)	754 (Q_1:649,5; Q_3:897,0)	722 (Q_1:486,0; Q_3:878,0)	667,5 (Q_1:514,0; Q_3:859,5)	3,37	0,19
Stapedial reflex 0,5 kHz	85 (Q_1:80; Q_3:90)	85 (Q_1:80; Q_3:91,25)	90 (Q_1:88,75; Q_3:100)	15,19	**<0,001**
Stapedial reflex 1 kHz	85 (Q_1:80; Q_3:90)	85 (Q_1:85; Q_3:90)	95 (Q_1:90; Q_3:100)	20,66	**<0,001**
Stapedial reflex 2 kHz	85 (Q_1:80; Q_3:88,75)	85 (Q_1:80; Q_3:86,25)	90 (Q_1:85; Q_3:95)	17,83	**<0,001**
Stapedial reflex 4 kHz	85 (Q_1:80; Q_3:90)	85 (Q_1:80; Q_3:91,25)	95 (Q_1:90; Q_3:100)	22,27	**<0,001**
